# Optimization and Transfollicular Delivery of Finasteride-Loaded Proniosomes for Hair Growth Stimulation in C57BL/6Mlac Mice

**DOI:** 10.3390/pharmaceutics13122177

**Published:** 2021-12-17

**Authors:** Wandee Rungseevijitprapa, Panikchar Wichayapreechar, Bhagavathi Sundaram Sivamaruthi, Damrongsak Jinarat, Chaiyavat Chaiyasut

**Affiliations:** 1 Department of Pharmaceutical Chemistry and Technology, Faculty of Pharmaceutical Sciences, Ubon Ratchathani University, Ubon Ratchathani 34190, Thailand; panikchar.wi@up.ac.th (P.W.); damrongsak.ji.56@ubu.ac.th (D.J.); 2 Department of Cosmetic Science, School of Pharmaceutical Sciences, University of Phayao, Phayao 56000, Thailand; 3 Innovation Center for Holistic Health, Nutraceuticals and Cosmeceuticals, Faculty of Pharmacy, Chiang Mai University, Chiang Mai 50200, Thailand; Sivamaruthi.b@cmu.ac.th; 4 Office of Research Administration, Chiang Mai University, Chiang Mai 50200, Thailand

**Keywords:** finasteride, proniosomes, response surface methodology, transfollicular delivery, hair growth, skin irritation, C57BL/6 mice, 5α-reductase inhibitor

## Abstract

The study aimed to develop the finasteride-loaded proniosome (FLP) to enhance the transfollicular delivery of finasteride (FN). The response surface methodology (RSM) combined with central composite design (CCD) with three independent variables (FN concentrations, total lipid content, and cholesterol content) was used to optimize the FLP preparation. The particles size, zeta potential, entrapment efficiency, and drug loading capacity of the FLP were analyzed. The transfollicular delivery of the optimum formulation was investigated in vitro. In vivo hair growth stimulation study was performed on C57BL/6Mlac mice dorsal areas. The Draize primary skin irritation test for erythema and edema was performed in the New Zealand white rabbit skin. The optimum FLP consists of 5.0 mM of FN, 10.1 mM of total lipid content, and 50.0% of the cholesterol in the total lipid. The prepared proniosome delivered the FN significantly (*p* < 0.05), compared to the naked finasteride solution in a dose- and time-dependent manner. The FLP treatment significantly increases the number and size of hair follicles in a dose-dependent manner. The efficiency of 1% FLP was comparable to the 2% minoxidil solution. The FLP exhibited no skin irritation after 72 h. Therefore, the results demonstrated that the FLP could stimulate hair growth via a transfollicular delivery system.

## 1. Introduction

Finasteride (FN) is approved as the first specific competitive inhibitor of 5α-reductase for the treatment of benign prostatic hyperplasia (BPH) and androgenic alopecia (AA) [1,2,3]. Several reports suggest that male pattern hair loss or AA are linked to dihydrotestosterone (DHT) [4,5]. DHT on the hair follicles was metabolized from testosterone by 5α-reductase [6]. Two isoforms of 5α-reductase are Type I and Type II, the former present in the skin and outer root sheath of the hair follicles, and the latter most evident in the prostate, seminal vesicles, and inner epithelial root sheath of hair follicles. Type II 5α-reductase activity in the dermal papillae of hair follicles has been reported as the major cause of AA. FN is a 5α-reductase inhibitor with a higher affinity to the Type II isoenzyme. FN can block dihydrotestosterone (DHT) [7], decreasing DHT activity by inhibiting the enzyme. FN is a 4-aza-3-oxosteroid compound classified in the Biopharmaceutical Classification System as a class II drug with high permeability and poor aqueous solubility. It was commercially available, and the FDA approved it only as an oral tablet of 1 and 5 mg to treat BPH and AA. FN may cause side effects, such as decreased libido and impotence [1]. Currently, topical use of FN is not commercially available, and the inhibition of type I 5α-reductase via the skin and outer root sheath of the hair follicles via the transfollicular delivery has yet to be investigated. Only topical minoxidil treatment (2% solution and 5% form) [7] is approved by the United States (US) Food and Drug Administration for the treatment of AA. Minoxidil belongs to a class of drugs known as vasodilators, which normalizes hair follicles and increases blood flow to the scalp, resulting in improved hair growth. However, topical minoxidil has not cured the specific cause of AA like FN.

Furthermore, minoxidil alcoholic solution may cause side effects like mild scalp dryness and irritation. The development and use of an FN-based topical treatment may improve the management of AA; this could be considered an interesting alternative method to minimize side effects.

A transfollicular route is an effective way to treat skin problems. The hair and hair follicles cover around 1% of the skin. Still, hair follicles are a significant drug delivery site to treat several skin-related diseases because of their close network of blood capillaries [3,8]. Skin diseases such as acne, seborrhea, hirsutism, and androgenetic alopecia are secondary to the excess local activity of androgens, more specifically, DHT in hair follicles [9]. There are many formulations for trans-follicular delivery into hair follicles, such as liposomes and niosomes follicles [9], ethosomes [10], and lipoplex [11]. Still, this delivery system is physically unstable during storage in aggregation, fusion, leaking, or hydrolysis of encapsulated drugs. 

Proniosomes, made of cholesterol and nonionic surfactants like niosomes, are less time consuming, involve no specialized equipment for the preparation, and are more stable during storage [12]. Niosomes are the proven carriers to deliver the bioactive compounds to the targeted cells or systems [13,14]. Niosomes can be prepared from the proniosomes by adding the aqueous phase of the drug to the proniosomes, with brief agitation at a temperature greater than the mean transition phase temperature of the surfactant [15,16,17]. In the present study, the coacervation method was used to prepare and optimize the FLP, as the method is simple, involves no specialized equipment, and is easy to scale up [14,18]. Porcine skin was selected to investigate transfollicular delivery since the porcine ear skin has similar histological characteristics to human skin [19]. Central composite design (CCD) was applied to the relevant independent variables [20,21]. This experimental design can reduce the number of experiments required, improve the optimum conditions’ accuracy, and enable the interaction between the test independent and dependent variables [21,22,23]. Proniosomes were prepared to achieve highly effective topical administration of FN, and CCD was used to optimize variables. The effect of FLP on hair growth of C57BL/6 mice was investigated by measuring the hair follicle morphology to evaluate its potential topical use for pharmaceuticals and cosmeceuticals.

## 2. Materials and Methods

### 2.1. Materials

Finasteride (Pharma Nueva; Chatuchak, Bangkok, Thailand), 95% ethanol (Italmar; Bangkok, Thailand), acetonitrile and cholesterol (Carlo Erba Reagents SpA; Cornaredo, MI, Italy), sorbitan monostearate (span 60) (Sigma-Aldrich Inc.; Louis, MO, USA), minoxidil (Johnson & Johnson; Bangkok, Thailand), porcine skin and abdominal part of stillborn pigs (Faculty of Agriculture, Ubon Ratchathani University; Ubon Ratchathani, Thailand), and C57BL/6Mlac mice and New Zealand white rabbit (National Laboratory Animal Center, Mahidol University; Nakhon Pathom, Thailand) were purchased from the respective companies and Universities. 

### 2.2. Preparation of Proniosomes

**Figure 1:**
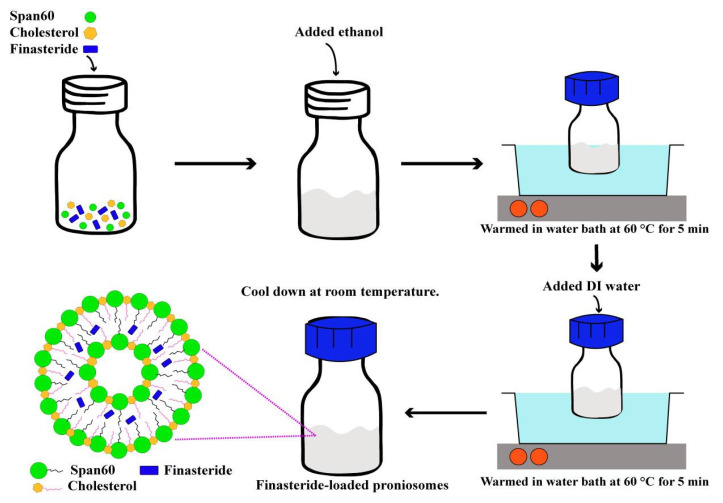
The schematic representation of the preparation of FLP.

**Figure 2:**
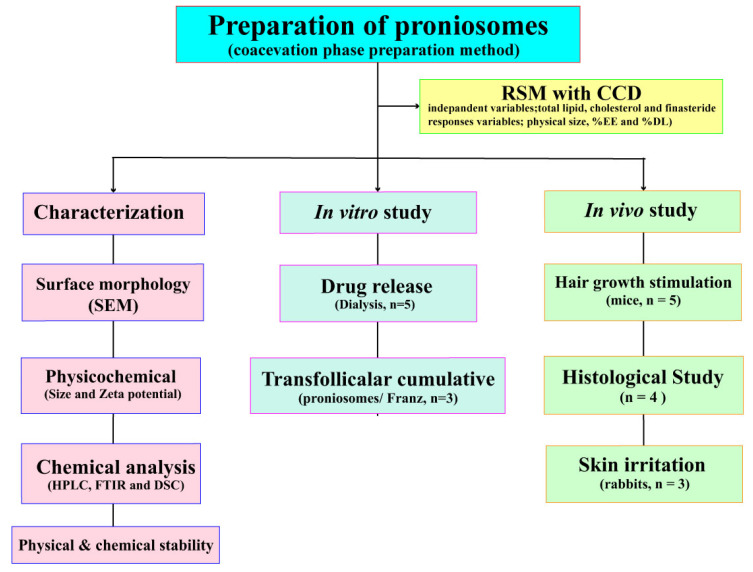
The flow diagram detailing the methodology of the study.

Proniosomes were prepared by the modified coacervation phase separation method (Figure 1) described by Shah et al. [24]. Briefly, various mixtures of span 60/cholesterol (Total lipid 10–30 mM), cholesterol (30–50%), and FN concentrations (1.5–5 mM) were accurately weighed (Table S1) and dissolved in 5 mL absolute ethanol. The mixture was warmed in a water bath (Julabo SW23, JULABO GmbH; Seelbach, Germany) at 60 °C for 5 min. Then, 1 mL of deionized water was added, and the mixture was kept in the water bath until the clear solution formed. The mixture was allowed to cool down to room temperature till the dispersion was converted to proniosomes. The FLP with varying FN concentrations (0.1, 0.5, and 1% *w*/*w*) were prepared. The prepared proniosomes were studied. The optimum FLP was investigated further to induce the hair growth study. The components of FLP are listed in Table S2 (Supplement file), and the study’s methodology is illustrated in Figure 2.

### 2.3. Experimental Design

A 3-factor, 3-level response surface methodology (RSM) combined with central composite design (CCD) using Design-Expert^®^ version 10 software (Stat-Ease; Minneapolis, MN, USA) was performed to explore the influence of relevant independent variables on physicochemical of FLP and to determine the optimal conditions. The three selected independent variables, such as FN concentrations (X_1_, 1.5–5.0 mM), the total lipid content of proniosomes (X_2_, 10–30 mM), and cholesterol in total lipid (X_3_, 30–50%), were considered. The ranges of each relevant variable were chosen based on the previous literature [25,26] since they affected the physicochemical properties of FLP. Other components used in the formulations were fixed constant (Table 1). Three-level test factors were coded as −1, 0, and +1 with −α and +α to generate the twenty randomized experiments with six replications at a central point. The studied response variables were the mean particle size of proniosomes (Y_1_), entrapment efficiency (Y_2_), and drug loading capacity (Y_3_). A multiple regression through the least square method was used to analyze the experimental data. Additionally, principal component analysis (PCA) and hierarchical cluster analysis (HCA) were performed to distinguish the initial dataset of the responses of the formed niosomes.

### 2.4. Desirability Function 

The desirability function was applied to select the best formulation. The FLP should accomplish the least particle size, while entrapment efficiency and drug loading capacity should attain maximum values (Table 1). The desirability value was calculated by Equation (1) [27,28].

(1) $$d_{i}=\{0\;{\hat{y}}_{i}\leq y_{i\;min}\;\left[\frac{{\hat{y}}_{i}-y_{i\;min}}{y_{i\;max}-y_{i\;min}}\right]\;y_{i\;min}<{\hat{y}}_{i}<y_{i\;max},\;for\;i=1,\;2,\;\ldots ,\;k\;1\;{\hat{y}}_{i}\geq y_{i\;max}\;$$

where $d_{i}$ is the desirability value of each response, $y_{i\;min}$ is the minimum acceptable value of ${\hat{y}}_{i},$ and $y_{i\;max}$ is the maximum acceptable value of ${\hat{y}}_{i}$. The obtained individual desirability was combined by applying the geometric mean to calculate the overall desirability (*D*).

(2) $$D={\left(d_{1}\times d_{2}\times d_{3}\right)}^{1/3}$$

The selected conditions were validated with three independent replications; the values obtained were compared to the predicted values. The t-test determined the significance of the results.

### 2.5. Characterization of Proniosome

#### 2.5.1. Surface Analysis 

The surface morphology of the proniosome was analyzed using a JSM-6010LV scanning electron microscope (JEOL Ltd.; Tokyo, Japan), operated at a voltage of 15.0 kV. The sample was prepared by dropping it on the glass slide and dried using the desiccator for 24 h. Then, the sample was coated with a thin layer of gold before the microscopic examination.

#### 2.5.2. ATR-FTIR Examination

The attenuated total reflection-Fourier transform infrared spectra (ATR-FTIR) was used to evaluate the FLP interaction. The samples were analyzed at the spectra of 400 to 4000 cm^−1^, and 2 cm^−1^ of the resolution were used in FT-IR spectrophotometer (Thermo Scientific, Nicolet 6700, Waltham, MA, USA) [29].

#### 2.5.3. Differential Scanning Calorimetry (DSC) Analysis

The proniosome suspensions were concentrated by centrifugal filtration (Amicon ultra-0.5, Merck; Darmstadt, Germany) 6000 rpm for 30 min at 4 °C. The pellet was collected and dried in a desiccator overnight. The FLP and pure FN were thermal scanned by Mettler DSC 821e (Mettle Toledo; Gießen, Germany) from 30 to 280 °C at a rate of 10 °C/min to cover the finasteride melting point, while cholesterol and span60 were 25 to 95 °C at 5 °C/min [30]. The melting peaks were calculated using STARe analysis software.

### 2.6. Analysis of the Dependent Variables

#### 2.6.1. Particle Size and Zeta Potential

The dynamic light scattering technique was used to measure the particle size of each sample by Nano ZS Zetasizer 90 (Malvern Instruments; Worcestershire, UK). The samples were diluted with deionized water at 1:300 (*v/v*) before measuring to achieve suitable optical density. The polydispersity index (PDI), the width of particle size, was determined. 

The electrophoretic light scattering technique measured the zeta potential. The time-dependent on the scattered light intensity of each formulation was determined at a scattering angle of 173° and performed at 25 °C. All data were evaluated using Zetasizer version 7.02 software.

#### 2.6.2. Entrapment Efficiency (EE) and Drug Loading Capacity (DL)

The centrifugation method determined the entrapment efficiency and drug loading capacity of FN in proniosomes. Unentrapped FN was separated by ultracentrifugation at 50,000 rpm for 1 h (OptimaTM L-100K; Beckman, UK). The upper layer (clear solution) was filtered through a 0.45 µm filter, and drug concentration was analyzed using high-performance liquid chromatography (HPLC, Waters Corporation; Milford, CT, USA). The percentage of entrapment efficiency and drug loading capacity was determined using Equations (3) and (4) [31,32].

(3) $$EE\;\left(\%\right)=\frac{\left(Total\;finasteride-Free\;finasteride\right)}{Total\;finasteride}\times 100$$

(4) $$DL\;\left(\%\right)=\frac{\left(Total\;finasteride-Free\;finasteride\right)}{Total\;finasteride-Free\;finasteride+Total\;lipid}\times 100$$

### 2.7. HPLC Analysis

The FN in proniosomes was quantified by the HPLC method. HPLC water breeze system (waters 2487 dual absorbance detector, 1525 binary HPLC pump, 717 plus autosampler) was used. C18 column (4.6 mm × 150 mm, 5 µm particle size) was used for the separation. 20 µL of the sample was injected. The acetonitrile and DI water (60:40) was used as a mobile phase with isocratic elution at a 1 mL/min flow rate. The UV detector was used at 210 nm.

### 2.8. In Vitro Transfollicular Delivery by Franz Diffusion Cells

#### 2.8.1. Skin Sample

Full-thickness skin from the dorsal area of stillborn pig’s skin was obtained from the farm of Faculty of Agriculture, Ubon Ratchathani University, Thailand. The pinching method removed the excess subcutaneous fat from shaved skin. For future studies, the porcine skin was rinsed with PBS (pH 7.4) and kept at −20 °C. The thickness of the porcine skin was 1.52 ± 0.13 mm.

#### 2.8.2. Preparation of Porcine Skin

The follicular closing technique blocked the porcine skin’s hair follicles [3]. Concisely, the number of hair follicles was counted using a microscope. A gauge needle blocked the follicular orifices of hair follicles with a small amount of nail varnish (Revlon Inc.; New York, NY, USA). They were dried for 5 min to close the follicular shunt completely. The blocked and unblocked hair follicle skins were tested by Franz diffusion cells. A sample with an equal number of hair follicles was used in the study.

#### 2.8.3. Transfollicular Cumulation

Transfollicular cumulation of stillborn pig’s skin was evaluated through the modified vertical Franz diffusion cells method [33]. The blocked and unblocked skins (3.5 cm^2^) were mounted with the viable epidermis side facing upwards to the donor compartment while the dermal side was in contact with the receiver. The receptor compartment was a mixture of phosphate-buffered saline (PBS, pH 7.4) and ethanol (ratio of 1:1). One mL of the FLP and finasteride solutions (FS) was placed in the donor compartment and covered with paraffin film. 500 µL of samples were removed and replaced with an equal volume from the receptor compartment. The experiments were performed in triplicate. The transfollicular cumulation was calculated by the following Equation (5).

Trans follicular cumulation (%) = % Full skin cumulation − % Follicle blocked skin(5)

### 2.9. In Vitro Release of Finasteride

FLP was evaluated for their drug release behavior by the dialysis method with slight modification [34]. The dialysis membranes (molecular weight cut off 12,000–14,000 Da, Sigma; St. Louis, MO, USA) were hydrated with deionized water overnight before the experiments. The FLP and 5.0 mM FS were placed into a dialysis bag and closed at both ends. The dialysis bag was immersed into a bottle containing 20 mL of release medium (phosphate buffer solution pH 7.4 and ethanol at a ratio of 1:1). This bottle was placed inside an incubator shaker at 32 ± 2 °C, 100 rpm. Aliquots of 1 mL release medium were withdrawn from the release medium at 1, 2, 4, 6, 8, 12, 24, and 48 h and replaced with equal fresh medium (*n* = 5). The FN release from proniosome was analyzed by HPLC. 

The in vitro drug release plots were fitted with the following kinetic models: zero-order, first-order, Korsmeyer-Peppas, and Higuchi, using Origin Pro 2018^®^ software (Microcal Software; Northampton, MA, USA) to calculate the correlation coefficient [35].

### 2.10. Stability Study

The stability of FLP was determined by storing it in different storage conditions, such as at 4, 25, and 40 °C, with 75% relative humidity for 4 months. The percentage of finasteride, particle size, and zeta potential were assessed every month. The results were compared to the baseline values.

### 2.11. Animal Studies

C57Bl/6Mlac mice (7 weeks-old male) and New Zealand white rabbits were purchased from the National Laboratory Animal Center, Mahidol University (Phutthamonthon, Nakhon Pathom, Thailand). The animals were maintained and fed with a standard laboratory diet and water ad libitum. The animals were housed with a 12:12 h light and dark cycle in an air-conditioned room at least 7 days before the experiment. The room temperature (23 ± 2°C) and humidity (~60%) were controlled automatically. The Animal Research Ethical Committee of Ubon Ratchathani University, Thailand, approved the study protocol (protocol no. 2/2555/Thesis).

### 2.12. Hair Growth Study

Thirty C57Bl/6Mlac mice were divided into six groups (*n* = 5 in each group). Blank proniosomes and 2% minoxidil solution served as the negative control and positive control, respectively. 1% FS, 0.1%, 0.5%, and 1% FLP were used as treatment groups. The mice’s dorsal area (2 × 4 cm^2^) was shaved with electric hair clippers. Briefly, 0.1 mL of each formula was applied once a day to the dorsal area for 4 weeks. After applying the samples, the degree of hair growth was scored and photographed on days 7, 14, 21, and 28. The score of hair growth was assigned as follows: score 0 = no hair growth observed, 1 = up to 20% of hair growth, 2 = 20–40% of hair growth, 3 = 40–60% of hair growth, 4 = 60–80% of hair growth, and 5 = 80–100% of hair growth. 

### 2.13. Histological Observation of Hair Follicles

The histological method was used to observe the hair follicles of mice [36]. All mice were sacrificed at the end of the hair growth study, and full-thickness skin biopsies were fixed in 10% buffered formalin. The skin biopsies (*n* = 4) were embedded in paraffin and stained with hematoxylin and eosin (H&E) for histopathological assessment under a light microscope (Olympus; Heidelberg, Germany).

### 2.14. Skin Irritation Test

The skin irritation test was performed in New Zealand white rabbits. The rabbits’ dorsal area (6 cm^2^) was shaved with electric hair clippers. Four test samples (1% FS, 0.1%, 0.5%, and 1% FLP) were applied to the marked dorsal area of the test animal. Blank proniosomes and 5% sodium lauryl sulfate (SLS) solution served as the negative and positive control. Gauze pads of about 1 × 1 cm^2^ were cut and used to apply 0.1 mL of each formula onto the dorsal skin sites. Each animal was covered with gauze and kept in a cage for 4 h. The patch was then removed after 1, 24, 48, and 72 h, and the degrees of erythema and edema were evaluated according to the Draize score. The primary irritation index indicates the level of skin irritation. 

### 2.15. Statistical Analysis

All data were expressed as mean ± standard deviation (SD). Data of two-group comparisons were analyzed for significance (*p* < 0.05) by independent t-test. Parametric data of more than two groups were analyzed by one-way ANOVA followed by Bonferroni’s multiple comparison. Non-parametric data of more than two groups were analyzed by the Kruskal-Wallis test. 

## 3. Results 

### 3.1. Effect of Proniosome Variables and Analysis of Variance

FLP was developed using RSM combined with CCD. The influence of FN concentration (X_1_), total lipid (X_2_), and cholesterol proportion in total lipid (X_3_) on the three independent factors [particle size in diameter (Y_1_), entrapment efficiency (Y_2_), and drug loading capacity (Y_3_)] were investigated (Table 2). In all experiments, the size of the particle was in the range of 220–346 nm in diameter, and the encapsulation efficacy (EE) was about 71–95%, with the drug loading capacity of 1.2–43%.

All the designed quadratic experimental models displayed significant outputs based on the coefficient of determination (R^2^) and adjusted R^2^ value (close to 1), which indicates association between the formulation and independent variables. All predicted R^2^ values were not lower than the adjusted R^2^ values, which indicated that the models provided a good response prediction (Table 3). The predicted responses in this experimental design were highly correlated with the actual values. In addition, adequate precision determined the signal-to-noise ratio greater than 4, which indicated that the experimental model was appropriate.

The fitted model for all target responses revealed the significant effect of independent variables (Figure 2) (Table 4).

Vesicle size = 468.00 − 8.98X_1_ + 0.98X_2_ − 7.03X_3_ + 0.53X_1_X_2_ + 0.61X_1_X_3_ + 0.02X_2_X_3_ − 4.08X_1_^2^ − 0.02X_2_^2^ + 0.02X_3_^2^

**Figure 3:**
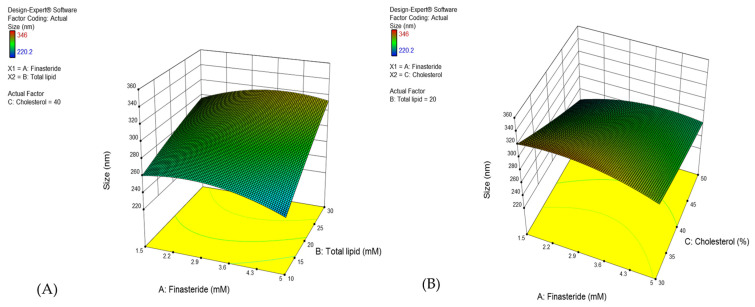

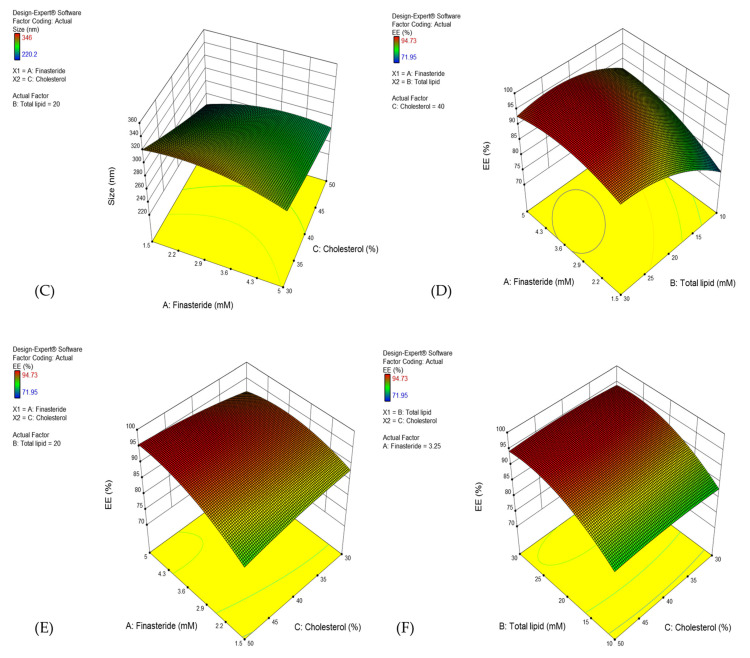

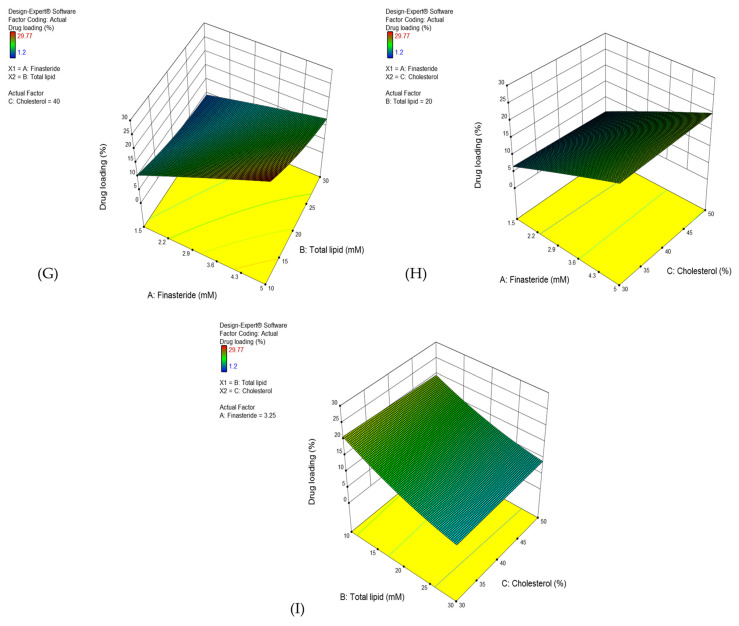
The 3-D surface response plots demonstrate the effect of FN concentration, total lipid content, and cholesterol proportion in total lipid on the vesicle size (**A**–**C**), % Entrapment efficiency (**D**–**F**), and % Drug loading capacity (**G**–**I**) of FLP.

The factors affecting the vesicle size are presented in Table S3 and Figure 3. The lipid (X2) and cholesterol (X3) concentrations affect the formulation. Moreover, all interactions (X_1_X_2_, X_1_X_3_, and X_2_X_3_) and quadratic terms (X_1_^2^, X_2_^2^, and X_3_^2^) were significant. The correlations between variables and vesicle size were obtained from regression analysis.

The drug loading capacity indicates the ability of the carrier to hold the drug effectively, which depends on the carrier that affects the drug physicochemical properties.

The optimal FLP was composed of 5.0 mM of FN, 10.1 mM of total lipid content, and 50.0% of cholesterol proportion in the total lipid. The expected and observed value difference was less than 5%, indicating that the optimal proniosomes formulation can be used in further experiments.

### 3.2. Characterization of FLP

#### 3.2.1. Surface Analysis by SEM

**Figure 4:**
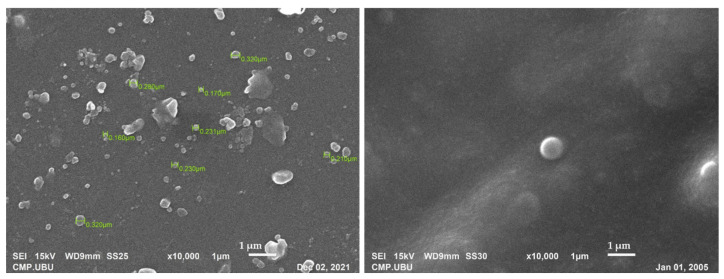
The representative SEM images of FLP at the magnification of 10,000×. Scale bar = 1 µm.

The prepared niosomes were visualized by SEM (Figure 4). It was found that niosomes have a uniform spherical morphology with a smooth surface. The particle size of the niosomes was in the range of 170–320 nm.

#### 3.2.2. ATR-FTIR Spectroscopy

ATR-FTIR spectroscopy was used to study the chemical structures of pure drug, cholesterol, span 60, and the probable interaction of the finasteride with the niosomal components (Figure S1, Supplementary Materials). 

All spectra display absorption bands at 2850–2917 cm^−1^ ascribed to the C–H stretching in alkyl groups. The ATR-FTIR spectra of FN demonstrated the characteristic peaks at 1666 and 1600 cm^−1^, corresponding to the two amide groups [37]. The characteristic broad bands of cholesterol at 3233 cm^−1^ are attributed to the O–H stretching. Other peaks at 3000–2850 cm^−1^, 1448 cm^−1^, and 1126 cm^−1^ correspond to C–H stretching, C–H bending, and C–O stretching, respectively [29]. The absorption bands of span 60 are observed at 3500 cm^−1^ (O–H stretching), 2937 cm^−1^ (–CH– stretching), and 1686 cm^−1^ (C=O stretching) [38].

#### 3.2.3. DSC Analysis

Thermogram of pure cholesterol, span 60, and FN were sharp endothermic peaks, as their melting points were detected at 39.15, 55.30, and 256.65 °C (Figure S2A). On the other hand, the thermogram of FLP (Figure S2B) displayed a single peak, with an approximate melting point of 50 °C.

### 3.3. In Vitro Transfollicular Delivery

**Figure 5:**
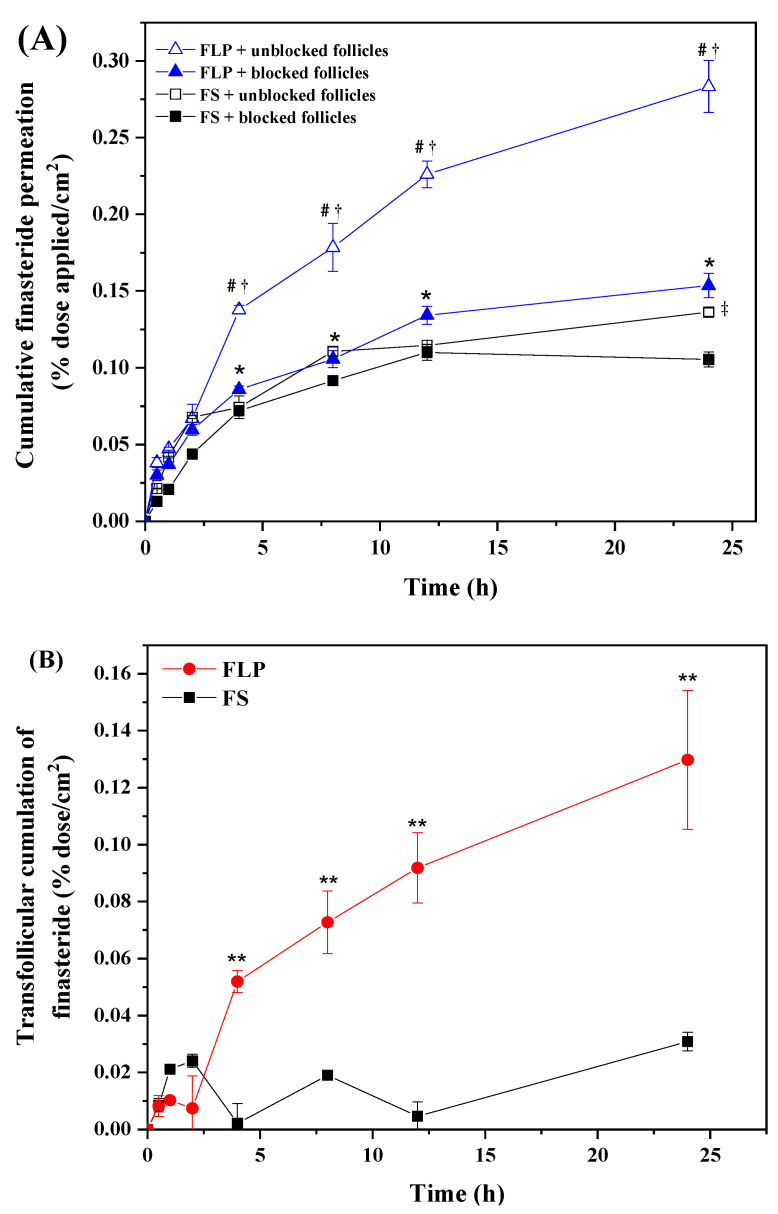
The cumulative amounts of FN in porcine skin by follicular closing technique (**A**) and calculated transfollicular accumulation (**B**). # *p <* 0.05 compared between FLP and FS unblocked follicles’ cumulation; † *p <* 0.05 compared between unblocked and blocked follicles’ cumulation of FLP; * *p <* 0.05 compared between FLP and FS blocked follicles’ cumulation; ‡ *p <* 0.05 compared between unblocked and blocked follicles’ cumulation of FS; ** *p <* 0.05 compared between FLP’s and FS’s cumulation.

In vitro transfollicular delivery was tested in blocked and unblocked (hair follicles) skin. The cumulative FN penetration through the pig’s skin was determined for 24 h. The optimized FLP (5 mM or 0.186%) and FS were used for the experiment. The cumulative penetration of FLP into unblocked and blocked hair follicles skin was divided into the initial (0–2 h) and terminal (4–24 h). The comparison of FN permeability through the transfollicular route between FLP and FS is represented in Figure 5. The cumulative permeation of FLP into the blocked and unblocked hair follicles skin was higher than the FS at the terminal phases (Figure 5A). The higher FLP was observed in the receptor medium, compared to the FS, which demonstrated increased transfollicular delivery of FLP. After 24 h, the transfollicular permeability of FS and FLP were recorded as 0.57 ± 0.06 and 2.41 ± 0.45 µg/cm^2^, respectively (Figure 5B).

### 3.4. In Vitro Drug Release

**Figure 6:**
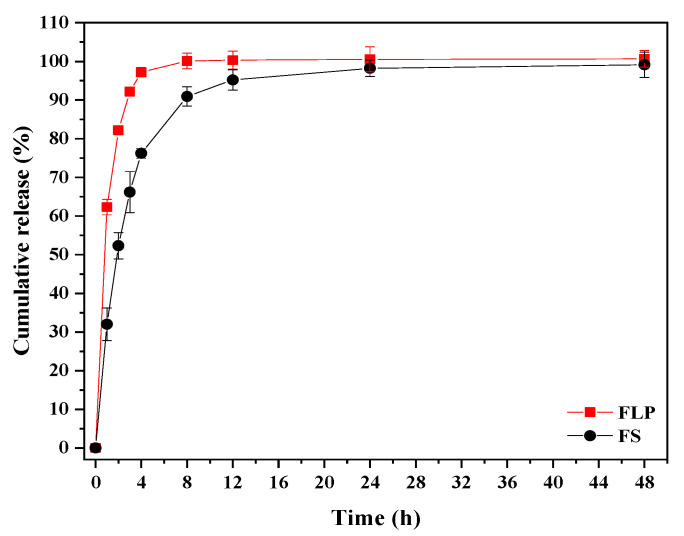
The cumulative release of FN from FLP and FS.

In vitro release of FN from proniosome was divided into the initial phase (0 to 4 h) and terminal phase (8 to 48 h). The release rate of FS was faster in the initial phase than FLP. The FS was completely (100%) released within 12 h. In contrast, the FLP demonstrated 80% FN release after 4 h of incubation. Then, it was estimated that 98% of FN was released from the niosomes after 48 h of incubation (Figure 6). 

### 3.5. Drug Release Kinetics

**Figure 7:**
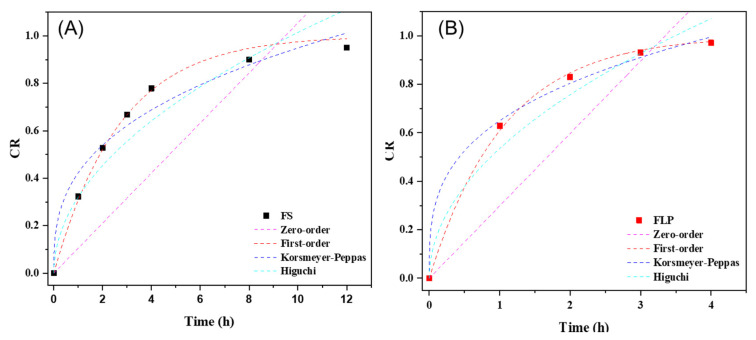
The kinetics release of FS (**A**) and FLP (**B**).

The kinetic release of FS and FLP are presented in Figure 7. The kinetic parameters of the release behavior of both samples are listed in Table 4. The first-order model provides the best correlation (R^2^ ≥ 0.99) to describe the drug release mechanism of both formulations. By considering the rate constant (k), it is found that the FS exhibits faster release than FLP.

### 3.6. Stability of FLP

The stability of FLP was evaluated (Table 5). The size and PDI of the FLP were found to increase with rising storage temperature and time. Moreover, the formulations can be considered stable since the loading capacity of the niosomes was not affected significantly (Figure S3). Though the encapsulation efficiency and loading capacity was reduced after storage, the niosomes are less sensitive to the storage time. 

### 3.7. Hair Growth Stimulation In Vivo

**Figure 8:**
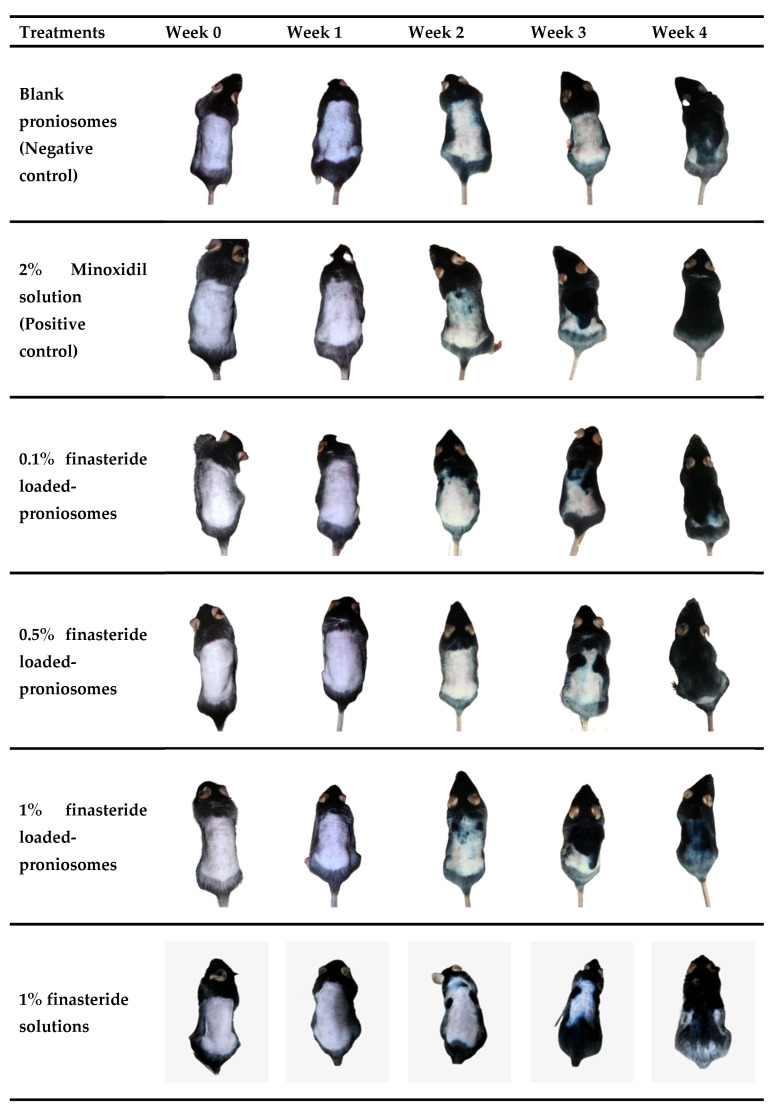
Photograph displaying the hair growth-promoting effects of various treatments in mice. The dorsal skins of 7-week-old C57BL/6 mice were shaved and topically applied with proniosomes (blank control), 2% minoxidil (positive control), 0.1%, 0.5% and 1% FLP and 1% FS, respectively.

The blank proniosomes and 2% minoxidil solution were used as negative control and positive control. 1% FS, 0.1%, 0.5%, and 1% FLP were used as experiment groups. The characteristics of hair growth patterns and hair growth-promoting scores of the experimental mice after 0, 7, 14, 21, and 28 days were presented in Figure 4 and Figure 5. As mentioned, the hair growth score was recorded every week to evaluate the hair growth-promoting activity of FLP. No sign of melanin formation was observed at the first week of the experiment, and the skin appeared pink (Figure 8).

After 14 days of treatment, the skin color was changed from pink to light grey. Slight hair regrowing signs were observed in 2% minoxidil, 1% FLP, and 1% FS groups. In contrast, there were no hair regrowing signs in the control group, and 0.1% and 0.5% FLP groups. 

Hair growth was observed in all the groups after 21 days of the treatment, with different degrees of hair growth. The 2% minoxidil group and 1% FLP group displayed more hair growth than other groups. 

Hair growth was observed in 20–40% of the experimental area in the control, and 0.1%, 0.5% FLP, and 1% FS treated experimental animals at 21 days of the study; whereas, the 2% minoxidil and 1% FLP group displayed hair growth around 40–60% of the experimental area.

After 28 days of the treatment, new hair growth was observed in all of the experimental groups. The hair growth area increased to 60–80%. In particular, the animals treated with 2% minoxidil and 1% FLP demonstrated the maximum (in almost 80% of the experimental area) hair growth (Figure 8).

**Figure 9:**
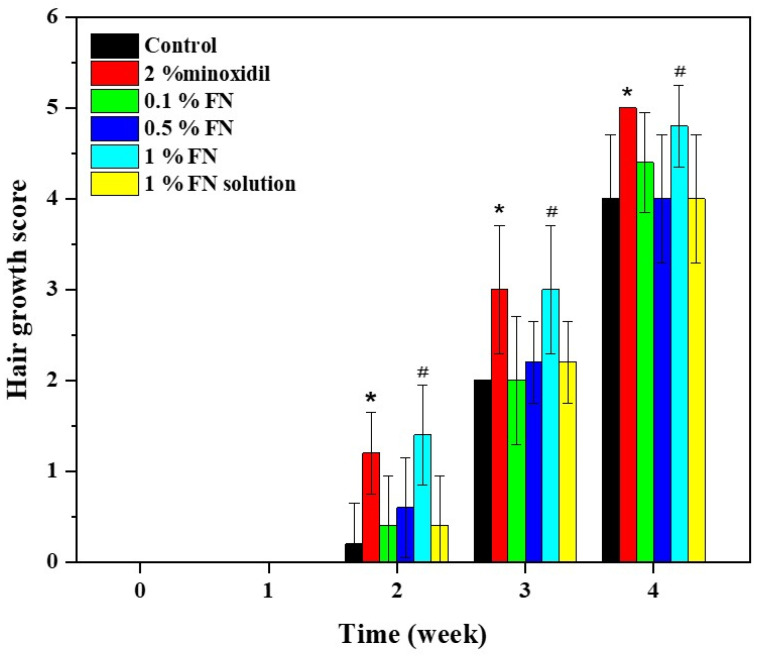
The hair growth scores of experimental animals. ^#,^* p < 0.05 compared to respective control groups.

The hair growth score of the 2% minoxidil, 1% FLP, 0.1% FLP, 0.5% FLP, 1% FS, and the negative control was 5, 4.8, 4.4, 4.0, 4.0, and 4.0, respectively (Figure 9). The hair growth-promoting scores of 2% minoxidil and 1% FLP treated groups were significantly higher than the control and other experimental groups (Figure 9). Significantly, the 1% FLP treatment increased the hair growth rate compared to the 1% FS. At week 2, the 2% minoxidil and 1% FN group displayed Prob = 0.0118 and Prob = 0.0048, respectively, compared to the control. At week three and four, the differences were Prob = 0.0163 and Prob = 0.0130 in 2% minoxidil group, respectively. The 1% FN treatment demonstrated the differences of Prob = 0.0163 and Prob = 0.0407 at week three and four, respectively.

### 3.8. Histological Observation of Hair Follicles

**Figure 10:**
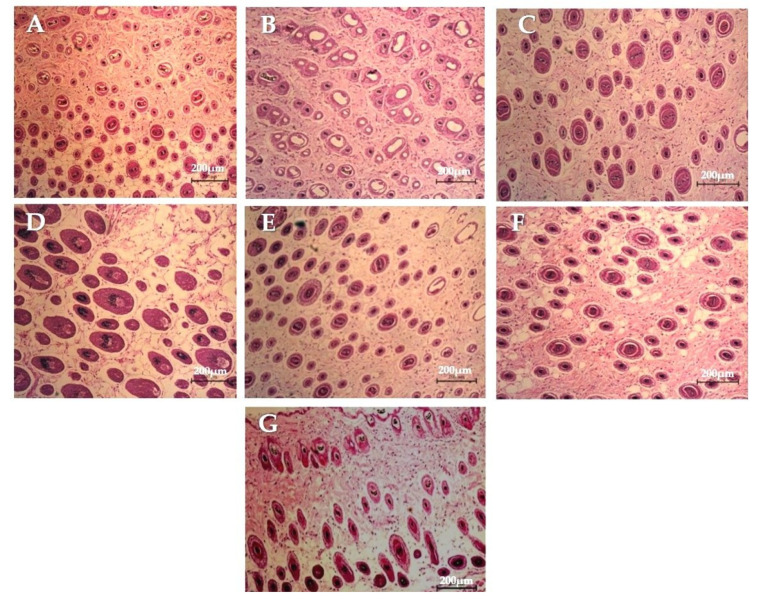
The microscopic images of histopathological examination of longitudinal skin sections treated with different test samples. 100× magnification. (**A**) No treatment; (**B**) blank proniosomes; (**C**) 2% minoxidil solution; (**D**) 0.1% FLP; (**E**) 0.5% FLP; (**F**) 1% FLP; (**G**) 1% FS. The scale bars = 200 µm.

The hair follicle counts (Table 6) and histopathological examinations (Figure 10) were reported. The results demonstrated that the FLP and minoxidil treatments increased the number and size of hair follicles compared to the control group.

FLP delayed the FN permeation at the same concentration as the naked FS. Still, the FLP were richly accumulated in the hair follicle route, resulting in an increased number of hair follicles and stimulated hair growth (*p* < 0.05). Mice treated with FLP and minoxidil were in the anagen phase of hair growth and displayed the maximum volume of dermal papilla cells (Figure 6).

### 3.9. Skin Irritation Test

The skin irritation potential of the developed formulations was assessed in New Zealand white rabbit skin by a Draize primary skin irritation test. The results of the skin irritation test are presented in Table 7. The prepared FLP displayed no erythema and edema scores compared to the controls. Skin irritation was scored and recorded according to the grades described by the primary irritation index (Table 8), which demonstrated that FLP caused no skin irritation on rabbit skin for 72 h.

## 4. Discussion

The proniosome was prepared by the coacervation phase separation method, an easy and reliable method to produce niosomes [39]. The size of vesicles is an important characteristic to penetrate the skin, which facilitates the delivery of the active compounds. A minimal size is more desirable, which can penetrate deeper skin. In contrast, the larger particles (around 600 nm in diameter) exhibit the deepest penetration and target hair follicles. However, penetration depths depend on particle type, size, and interaction between the drug and the sebum [40,41]. 

Furthermore, cholesterol is used for proniosome preparation because it can improve stability and increase the encapsulation due to its lipophilic behavior of the lipid bilayer, resulting in high loading capacity (Table S1). It was observed from the drug loading capacity equation that cholesterol content had a positive effect on drug loading concentration. Cholesterol can form hydrogen bonds with nonionic surfactants like Span 60 and increase drug encapsulation [42].

Additionally, the hair cycle and parts of the skin also affect the drug accumulation within hair follicles. Many researchers found that a diameter around 200 nm can be trapped in the hair follicle structure and penetrate through the sebum, resulting in effective delivery of the drug [43,44]. The size of the developed FLP was in the range of 170–320 nm (Figure 4).

In the present experiment design, the reduced total lipid concentration and increased cholesterol concentration influence the size of proniosome vesicles (Figure 3A–C). Hydrophobic surfactant (span 60) played a role in the reduced size of the vesicle due to its low hydration at the hydrophilic head [45]. However, a high amount of span 60 increases the viscosity in the formulations, which increases the vesicle or particle size [46]. On the other hand, cholesterol concentration affects the physicochemical properties of proniosomes, especially the vesicle bilayer stability [38]. Cholesterol can decrease the surface free energy at the bilayer and vesicle size [47]. The particle size of the niosomes may vary depending on the lipid concentration in the preparation [48].

The statistical analysis demonstrated that %EE was affected by the finasteride (X_1_) and total lipid concentration (X_2_). The quadratic analysis demonstrated X_1_ and X_2_, and the interaction of X_1_X_2_ and X_2_X_3_ influenced the EE. The resulting equation was as follows [28]:

%EE = 42.51 + 6.46X_1_ + 2.68X_2_ + 0.24X_3_ − 0.15X_1_X_2_ + 0.12X_1_X_3_ − 0.96X_1_^2^ − 0.04X_2_^2^ − 0.01X_3_^2^

As per the CCD model, a high concentration of total lipid and larger vesicle size may produce higher %EE (Figure 3A,C). The %EE was also directly affected by the FN concentration, and the optimum concentration was 4–4.5 mM (Figure 3D,E). FN is a hydrophobic compound with a partition coefficient (log *P*) of 3.0. Moreover, the proniosomes comprised of low hydrophilic-lipophilic balance (HLB)-valued surfactant, span60 (4.7), which improved the percentage of entrapment [49]. The higher concentration of finasteride enables the increased entrapment of FN [50,51]. The cholesterol quantity in total lipid did not affect the %EE (Figure 3E,F) since it proved that the use of span 60 as a surfactant improves the vesicle’s physicochemical property, which helps deliver the lipophilic drugs effectively [42]. 

Previously, Ahmed and Rizq [48] reported that nano-transferosomal gel formulations prepared with different molar concentrations of Phospholipon 90G (P90G) affected the EE. In detail, the formulations containing 1:2.0, 1:2.75, and 1:3.5 molar ratios of FN and P90G exhibited ~69.72, 89, and 93% of EE, respectively [47]. We reported that the EE was about 70–95%, indicating that proniosomes effectively carry the FN (Table 4). The optimal FLP was used in further experiments.

Drug loading capacity indicates the ability of the carrier to hold the drug effectively, which depends on the carrier that affects the drug physicochemical properties.

The statistical analysis demonstrated (Table S3) that all study factors significantly support the drug loading efficacy, except the quadratic term of cholesterol concentration (X_3_^2^), as per the following equation.

%DL = 14.69 + 6.29X_1_ − 0.86X_2_ − 0.17X_3_ − 0.14X_1_X_2_ + 0.02X_1_X_3_ − 0.06X_1_^2^ + 0.01X_2_^2^

Although the %DL value depended on FN and total lipid concentration (Figure 1G–I), the software demonstrated that the variables performed as per the predictable quadratic model. It suggested that span 60 was a suitable lipophilic surfactant to develop FLP.

An ATR-FTIR spectroscopy was used to study the probable interaction of the FN with the niosomal components (Figure S1).

All pure substances displayed similar peaks, as reported by [35,37], indicating the purity of samples and suitable for niosomal formulations. For the spectrum of niosomal formulations, most absorption peaks correspond to the characteristic peaks of pure ingredients. Most of the peaks are diffused, demonstrating a strong physical interaction between finasteride, cholesterol, and surfactants.

Thermal analysis was conducted to evaluate the ingredient interactions. The results revealed that ingredients in the proniosomes are compatible, with a slight difference in their melting points [52].

Pig’s skin has been widely used in various fields because of several histological and immunohistochemical similarities with human skin [53]. The efficiency of in vitro skin permeability did not significantly differ among the porcine and human skin [19]. In vitro transfollicular delivery was tested in blocked and unblocked skin. The delivery of the substances into the unblocked hair follicles skin may be possible through two main routes, such as transepidermal and transfollicular routes. In contrast, skin delivery occurs through only the transepidermal route in blocked hair follicles. Thus, the difference in infused flux between the unblocked hair follicles skin (transfollicular and transepidermal route) and the blocked hair follicles skin (transepidermal route) was calculated as the transfollicular penetration flux [3]. 

Recently, a novel method for assessing transfollicular penetration was developed by combining the dermal micro-dialysis and follicular closing technique, which is worth investigating transfollicular and intercellular skin penetration [54]. The main difference between the combined technique and Franz diffusion cell were skin area and experimental setup. The combined technique used full-thickness skin, but it monitors small skin around the dialysis membrane. Furthermore, the combined technique required direct contact with hair follicles to study the transfollicular penetration. In contrast, the Franz diffusion cell method uses the epidermis and dermis of the skin for the study. The skin area with blocked and unblocked hair follicles can be used to study the time-dependent transfollicular penetration. Therefore, the Franz diffusion cell method is more suitable for transfollicular studies [54]. 

Hydrophobic sebum, produced by the sebaceous gland, provides an easy way for the oil soluble-drug permeation, but it prevents the transfer of hydrophilic drugs or substances [55]. Proniosomes are hydrophobic, which facilitates the ease of diffusion with sebum and penetrates the hair follicles. In addition, the particle size of the FLP was in the range of 220–340 nm, which can be easily absorbed into the skin via hair follicles, since the skin’s pore size is 10–70 µm [56].

The results suggested that the FLP delayed drug permeation more than the FS, with better drug localization in the hair follicles (Figure 5). This result was consistent with the finding that liposome has higher permeation and effectively targets the drugs into the targeted site [57]. Furthermore, massage and occlusion can stimulate penetration of nanoparticles into the hair follicle through the transfollicular pathway because of the mechanism-like geared pump movement [58]. Interestingly, massage application positively affected the follicular penetration depth for a small particle size (around 300 nm) into ex vivo skin [59]. It has been suggested that massage application should increase the substance or nanoparticles penetration into the hair follicle.

The in vitro release study demonstrated that about 98% of FN was released from the FLP after 48 h, whereas within 12 h, complete release of free-finasteride was observed (Figure 6). The results indicated that incorporating FN in proniosome could enhance the slower release of the drug.

The in vitro drug release plots were fitted with the following kinetic models; zero-order, first-order, Korsmeyer-Peppas, and Higuchi, using Origin Pro 2018^®^ software (Microcal Software; Northampton, MA, USA) to calculate the correlation coefficient [35]. The kinetic release of FS and FLP are presented in Figure 7. The kinetic parameters of the release behavior of both samples are listed in Table 4. The first-order model provides the best correlation (R^2^ ≥ 0.99) to describe the drug release mechanism of both formulations. By considering the rate constant (k), it is found that the FS exhibits faster release than FLP. The results suggest that the formulation of proniosome can enhance the appropriate release of the drug. Thus, the proniosome formulation could be regarded as one of the effective alternatives for both transdermal delivery and topical delivery of FN. Moreover, the stability study revealed that the prepared FLP was stable for four months (Table 5; Figure S3).

C57BL/6mlac mice are commonly used to study hair growth-related studies, since the animal has a hair cycle and hair growth pattern similar to humans, with the difference in the hair cycle length [60,61]. Though the hair follicle dimensions differ in animals compared to human skin, no differences were found in the penetration depths of nanoparticles (~320 nm) between human skin and porcine ear skin [62]. The minoxidil-loaded niosomes displayed increased transfollicular bioavailability and attained superior cutaneous delivery, enhancing skin penetration and increasing the number of hair follicles [63]. The results of the current study revealed that the proniosomes enhanced the desired drug (FN) delivered to the targeted site, stimulating hair growth [17,64]. The hair growth stimulating the ability of the FLP was comparable to minoxidil in a dose and time-dependent manner (Figure 8 and Figure 9).

The FLP and minoxidil treatments increased the number and size of hair follicles compared to the control group (Table 6, Figure 10). All the tested concentrations of the FLP could stimulate hair growth from telogen to anagen phase [26]. Moreover, FLP treated animals displayed more hair follicles than the control in a dose-dependent manner. The 1% FLP treatment results were equivalent to a 2% minoxidil treatment, but the concentration of the studied drug (FN) was lesser than the minoxidil solution used. The results also indicated that proniosome based treatment needs less frequent dosing, which is one of the major advantages of drug delivery systems over traditional hair regrowing treatments. 

The improvement in transfollicular permeation of FN using proniosome has been evident in this study (Figure 5). At the same concentration, FLP delayed the FN permeation compared with the naked-FS. Mice treated with FLP and minoxidil were in the anagen phase of the hair growth and displayed the maximum volume of dermal papilla cells (Figure 10).

The presence of surfactants and alcohol in the formulation may cause slight irritation. The physical form of niosomes and proniosomes rarely cause irritation and toxicity in vivo due to the alkyl chain length and type of surfactant used. The high alkyl chain length of the surfactant could reduce toxicity compared with the short-chain. In addition, the surfactant type of niosomal formulation might affect keratinocyte cells, especially ether-type surfactants. However, in this study, span 60 (sorbitan ester) was used to prepare the proniosomes, nonionic surfactant, and biodegradable surfactant. The results of the skin irritation study proved that FLP was safe and did not cause skin irritation on rabbit skin (Table 7 and Table 8).

As stated earlier, the minoxidil-loaded niosomes treatment-induced hair growth [63]. The studies demonstrated that *Oryza sativa* bran extract-loaded niosome gel was safe and promoted hair growth [65]; Dithranol and dexpanthenol-loaded niosomes were also appropriate for the treatment of alopecia areata [66]. The meta-analysis of randomized controlled trials demonstrated that FN (1 mg) is effective for treating AA [67]. The present study detailed the preparation of optimum FLP and its efficacy in vitro and in vivo.

## 5. Conclusions

The results confirmed that using 5.0 mM of FN, 10.1 mM of total lipid content, and 50.0% of cholesterol could produce effective FLP. About 2.41 ± 0.45 µg/cm^2^ of FN was delivered successfully via the transfollicular route by the proniosomes. In the experimental mice model, the treatment with 1% FLP enhanced hair growth effectively in terms of the number and size of hair follicles. The prepared FLP did not cause any irritation in the skin. Accordingly, the results revealed that FLP could be used to inhibit the Type I 5α-reductase, which may aid in managing hair loss without any adverse side effects. Further studies are recommended to develop and commercialize the FLP-based hair loss preventive pharmaceutical agents.

## Figures and Tables

**Table 1:** Independent variables, symbols, coded levels, and actual values of the experimental design.

Independent Variable	Symbol	Code Levels				
		−*α*	−1	0	+1	+*α*
Finasteride concentration (mM)	X_1_	0.31	1.50	3.25	5.00	6.19
Total lipid content (%w/w)	X_2_	3.18	10.00	20.00	30.00	36.82
Cholesterol in total lipid (%mol/mol)	X_3_	23.18	30.00	40.00	50.00	56.81
**Dependent variables**		**Constraints**				
Mean particle size (nm)	Y_1_	Minimize				
Entrapment efficacy (%EE)	Y_2_	Maximize				
Drug loading capacity (%)	Y_3_	Maximize				

*α* = 1.682.

**Table 2:** Design matrix for the preparation of FLP using orthogonal design.

No. of the Experiment	Independent Variable (Design Level)			Dependent Variable		
	$X_{1}X_{1}\;(\mathrm{mM})$	$X_{1}X_{2}\;(\mathrm{mM})$	$X_{1}X_{3}\;(\%)$	Y_1_ (nm)	Y_2_ (%)	Y_3_ (%)
1	6.19	20.00	40.00	255.80 ± 4.09	90.31 ± 1.35	21.85 ± 1.52
2	1.50	30.00	50.00	263.40 ± 5.55	88.21 ± 1.42	4.22 ± 1.49
3	0.31	20.00	40.00	258.20 ± 3.77	79.77 ± 2.31	1.20 ± 1.34
4 ^c^	3.25	20.00	40.00	290.60 ± 3.05	93.68 ± 1.65	13.21 ± 1.32
5	1.50	10.00	50.00	220.20 ± 1.99	72.57 ± 2.01	9.81 ± 1.05
6 ^c^	3.25	20.00	40.00	290.60 ± 3.05	93.68 ± 1.65	13.21 ± 1.32
7 ^c^	3.25	20.00	40.00	292.20 ± 2.59	94.57 ± 3.74	13.32 ± 1.54
8 ^c^	3.25	20.00	40.00	295.00 ± 3.74	94.05 ± 1.66	13.25 ± 1.51
9	5.00	30.00	50.00	300.20 ± 5.40	94.73 ± 2.13	13.63 ± 1.31
10 ^c^	3.25	20.00	40.00	293.00 ± 3.67	93.35 ± 1.51	13.17 ± 1.26
11	3.25	20.00	56.82	285.60 ± 4.72	91.26 ± 1.12	12.91 ± 1.64
12 ^c^	3.25	20.00	40.00	292.60 ± 2.61	93.60 ± 1.33	13.20 ± 1.33
13	1.50	30.00	30.00	335.60 ± 8.20	92.01 ± 2.65	4.39 ± 2.33
14	3.25	3.18	40.00	242.80 ± 3.70	71.95 ± 1.24	43.05 ± 1.61
15	5.00	10.00	50.00	223.00 ± 1.81	89.36 ± 1.31	30.88 ± 1.23
16	5.00	10.00	30.00	261.20 ± 4.27	84.77 ± 3.01	29.77 ± 1.84
17	3.25	36.82	40.00	329.40 ± 6.69	91.61 ± 2.34	7.48 ± 1.31
18	5.00	30.00	30.00	332.40 ± 2.97	83.24 ± 2.61	12.18 ± 1.30
19	3.25	20.00	23.18	346.00 ± 1.86	95.44 ± 1.11	13.42 ± 1.67
20	1.50	10.00	30.00	261.60 ± 5.22	89.45 ± 1.32	11.83 ± 2.21

^c^, center point. Data are expressed as the means ± S.D (*n* = 3). X_1_, FN concentration; X_2_, total lipid; X_3_, cholesterol proportion in total lipid; Y_1_, particle size in diameter; Y_2_, entrapment efficiency; Y_3_, drug loading capacity.

**Table 3:** The regression analysis of 3-level 3-factor CCD of finasteride-loaded proniosomes.

Response	Model	R^2^	Adjusted R^2^	Predicted R^2^	Adequate. Precision	*p*-Value
Y_1_: Size	Quadratic	0.9987	0.9972	0.9857	84.4387	<0.001
Y_2_: %EE	Quadratic	0.9934	0.9850	0.8745	32.3427	<0.001
Y_3_: %DL	Quadratic	0.9999	0.9998	0.9989	494.4610	<0.001

Y_1_, Particle size in diameter; Y_2_, Entrapment efficiency (%EE); Y_3_, Drug loading capacity (%DL).

**Table 4:** The kinetic release behaviors of FS and FLP.

Formulation	Equation	Parameter	FS	FLP
Kinetic Model				
Zero-order	CR = Mt/Me = k^o^	k_0_ × 10^−2^	0.1055	0.2987
		R^2^	0.2747	0.6624
Frist-order	CR = 1 − e^−k1t^	k_1_ × 10^−2^	0.3678	0.9392
		R^2^	0.9943	0.9987
Korsmeyer-Peppas	CR = k_KP_t^n^	k_KP_ × 10^−2^	0.4229	0.6500
		n	0.3511	0.3072
		R^2^	0.9569	0.9957
Higuchi	k_H_t^0.5^	k_H_ × 10^−2^	0.3208	0.5355
		R^2^	0.9090	0.9623

**Table 5:** The size, PDI, and ZP of FLP stored at 4, 25, and 40 °C, 75%RH for 4 months (*n* = 4).

Month	Characteristics	Storage Condition		
		4 °C	25 °C 75%RH	40 °C 75%RH
0	Size (nm)	312 ± 5.97	325 ± 4.70	335 ± 5.44
	PDI	0.40 ± 0.04	0.46 ± 0.03	0.45 ± 0.02
	Zeta potential (mV)	−31.43 ± 0.97	−32.38 ± 1.33	−32.23 ± 1.32
1	Size (nm)	316 ± 3.87	321 ± 2.50	327 ± 4.22
	PDI	0.42 ± 0.05	0.38 ± 0.03	0.44 ± 0.04
	Zeta potential (mV)	−34.30 ± 3.92	−32.85 ± 1.91	−36.53 ± 1.78
2	Size (nm)	308 ± 4.99	315 ± 3.54	329 ± 4.38
	PDI	0.45 ± 0.02	0.43 ± 0.02	0.43 ± 0.08
	Zeta potential (mV)	−26.00 ± 3.12	−34.25 ± 1.40	−32.30 ± 1.53
3	Size (nm)	316 ± 2.05	323 ± 2.52	331 ± 0.25
	PDI	0.41 ± 0.05	0.43 ± 0.07	0.45 ± 0.03
	Zeta potential (mV)	−31.55 ± 1.82	−30.48 ± 0.75	−30.45 ± 1.48
4	Size (nm)	319 ± 5.45	327 ± 3.87	336 ± 3.22
	PDI	0.45 ± 0.03	0.46 ± 0.04	0.43 ± 0.07
	Zeta potential (mV)	−30.70 ± 0.75	−30.78 ± 0.90	−30.63 ± 0.42

**Table 6:** The hair follicle counts of minoxidil, FLP, and FS treated C57BL/6 mice.

Group	Hair Follicle Count
Controls	
No treatment	39.0 ± 1.41
Blank proniosome	36.0 ± 4.69
2% minoxidil solution	67.6 ± 2.70 ^a^
0.1% finasteride loaded-proniosomes	58.5 ± 3.53 ^a^
0.5% finasteride loaded-proniosomes	63.8 ± 3.63 ^a^
1% finasteride loaded-proniosomes	66.8 ± 6.22 ^a^
1% finasteride solution	60.2 ± 4.15 ^a^

^a^*p <* 0.05 compared with 1% FS. All data are represented as means ± SD of four biopsies per donor. All data are represented as means ± SD of four (*n* = 4) pictures.

**Table 7:** Skin irritation scores. FLP, Finasteride loaded-proniosomes.

Group	1 h		2 h		48 h		72 h	
	Erythema	Edema	Erythema	Edema	Erythema	Edema	Erythema	Edema
Gauze pad	0	0	0	0	0	0	0	0
5% SLS solution	1.5	0	1.5	0	1.25	0	1	0
Blank proniosomes	0	0	0	0	0	0	0	0
0.1% FLP	0	0	0	0	0	0	0	0
0.5% FLP	0	0	0	0	0	0	0	0
1% FLP	0	0	0	0	0	0	0	0

**Table 8:** Skin primary irritation index. FLP, Finasteride loaded-proniosomes.

Group	Primary Irritation Index	Class
Gauze pad	0	No irritation
5% SLS solution	0.65	Slight irritation
Blank proniosomes	0	No irritation
0.1% FLP	0	No irritation
0.5% FLP	0	No irritation
1% FLP	0	No irritation

## Data Availability

The data presented in the manuscript is available on request from the corresponding author.

## Supplementary Materials

The following are available online at https://www.mdpi.com/article/10.3390/pharmaceutics13122177/s1, Figure S1: ATR-FTIR spectra of proniosome, finasteride, cholesterol, and span 60, Figure S2: DSC thermograms of FLP and pure compositions, Figure S3: The impact of time and temperature during storage, Table S1: Components of finasteride-loaded proniosomes, Table S2: Components of finasteride-loaded proniosomes, Table S3: ANOVA for the responses of finasteride-loaded proniosomes in CCD.
