# Supplementary material for: Optimization and Transfollicular Delivery of Finasteride-Loaded Proniosomes for Hair Growth Stimulation in C57BL/6Mlac Mice
Source: Pharmaceutics. 2021 Dec 17;13(12):2177. doi: 10.3390/pharmaceutics13122177 (PMC8706991; doi:10.3390/pharmaceutics13122177)
Supplement: Supplementary file 1 [file pharmaceutics-13-02177-s001.zip › pharmaceutics-1465700-supplementary.pdf]

pharmaceutics

MDPI

Supplementary Materials: Optimization and Transfollicular Delivery of Finasteride-Loaded Proniosomes for Hair Growth Stimulation in C57BL/6Mlac Mice

Wandee Rungseevijitprapa, Panikchar Wichayapreechar, Bhagavathi Sundaram Sivamaruthi, Damrongsak Jinarat, Chaiyavat Chaiyasut

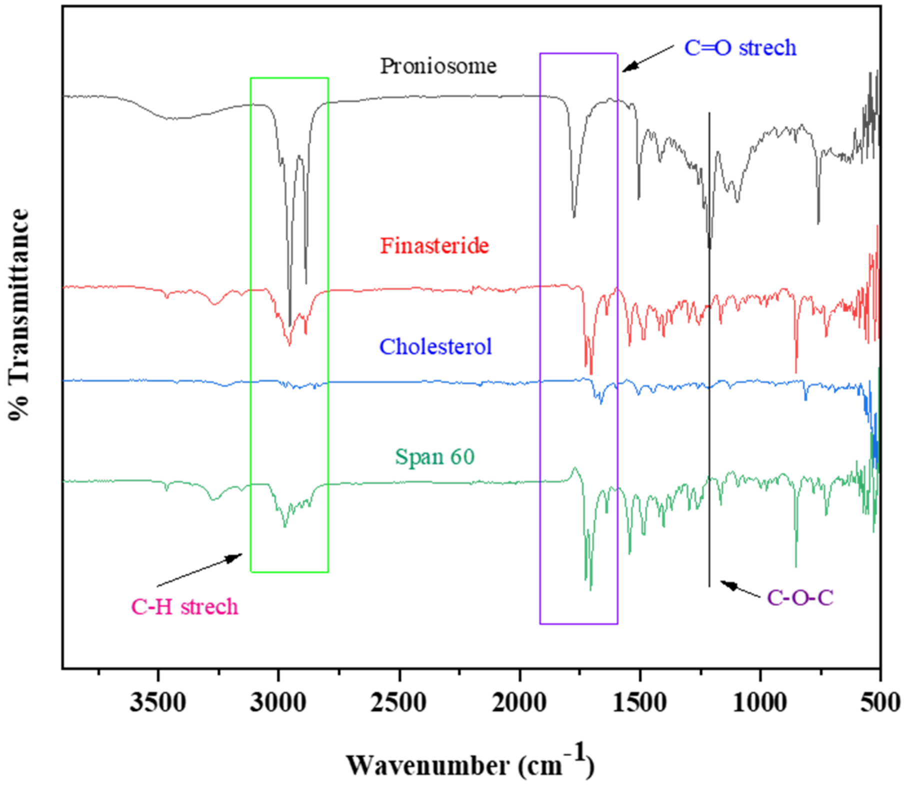

Figure S1. ATR-FTIR spectra of proniosome, finasteride, cholesterol, and span 60.

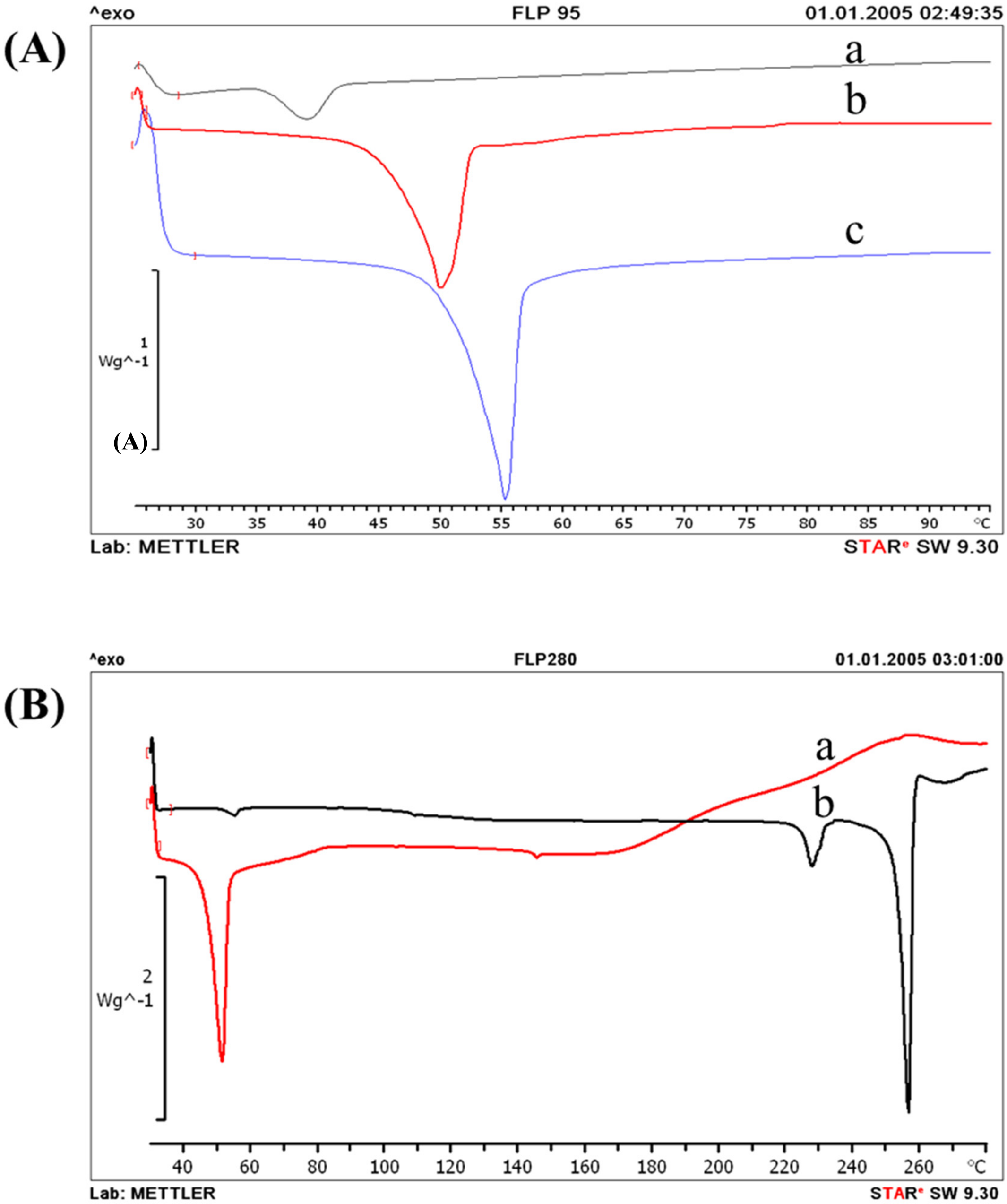

Figure S2. DSC thermograms of FLP and pure compositions: Figure (**A**): cholesterol a, FLP b and span 60 c scanned 25 – 95 °C; Figure (**B**): FLP a and bulk finasteride b scanned 30 – 280 °C.

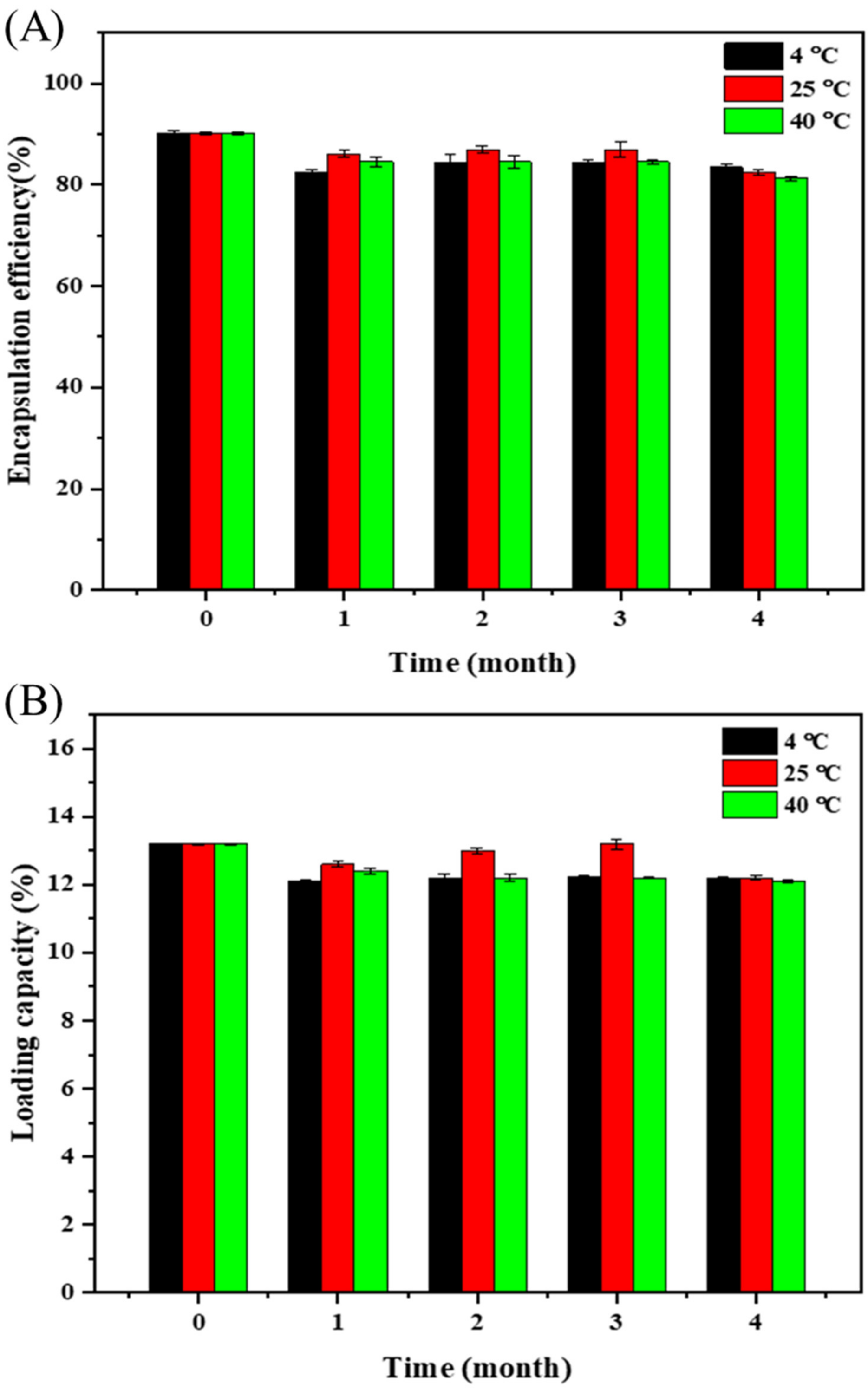

Figure S3. The impact of time and temperature during storage. (**A**) Encapsulation and (**B**) Loading capacity of FLP.

Table S1. Components of finasteride-loaded proniosomes.

No. of the experiment	Finasteride)mg(	Cholesterol)mg(	Span 60 (mg(
1	230.61	309.30	309.30
2	55.88	579.93	579.93
3	11.55	309.30	309.30
4	121.08	309.30	309.30
5	55.88	193.31	193.31
6	121.08	309.30	309.30
7	121.08	309.30	309.30
8	121.08	309.30	309.30
9	186.27	579.93	579.93
10	121.08	309.30	309.30
11	121.08	439.35	439.35
12	121.08	309.30	309.30
13	55.88	347.96	347.96
14	121.08	49.18	49.18
15	186.27	193.31	193.31
16	186.27	115.99	115.99
17	121.08	569.41	569.41
18	186.27	347.96	347.96
19	121.08	179.24	179.24
20	55.88	115.99	115.99

Table S2. Components of finasteride-loaded proniosomes. *phosphate buffer solution pH 7.4 and ethanol at ratio of 1:1.

Formulation	Finasteride (mg)	Cholesterol (mg)	Span 60 (mg)	Total lipid (mg)
0.1% finasteride loaded-proniosomes	7.76	197.18	219.62	416.8
0.5% finasteride loaded-proniosomes	31.05	197.18	219.62	416.8
1% finasteride loaded-proniosomes	62.09	197.18	219.62	416.8
1% finasteride solutions*	62.09	-	-	-

Table S3. ANOVA for the responses of finasteride-loaded proniosomes in CCD.

Response	Source	SS	Df	MS	*F*-value	*p*-value
**Mean**	Model	23467.5026	9	2607.5003	666.5085	0.0000[c]
**Particle**	X_1	6.6996	1	6.6996	1.7125	0.2270
**Size**	X_2	8114.7108	1	8114.7108	2074.2179	0.0000[c]
	X_3	5846.5125	1	5846.5125	14944391	0.0000[c]
	X_1X_2	490.3626	1	490.3626	125.3430	0.0000[c]
	X_1X_3	662.9122	1	662.9122	169.4458	0.0000[c]
	X_2X_3	28.4225	1	28.4225	7.2651	0.0273[a]
	X_1^2	2052.4421	1	2052.4421	524.6289	0.0000[c]
	X_2^2	63.6608	1	63.6608	16.2725	0.0038[b]
	X_3^2	40.7819	1	40.7819	10.4244	0.0121[b]

	Lack of fit	19.4241	3	6.4747	2.7266	0.1539[ns]
	Cor total	23498.8	17			
**%EE**	Model	843.6348	9	93.7372	117.4562	0.0000[c]
	X_1	96.8652	1	96.8652	121.3757	0.0000[c]
	X_2	241.6361	1	241.6361	302.7791	0.0000[c]
	X_3	0.1117	1	0.1117	0.1400	0.7193[ns]
	X_1X_2	28.4029	1	28.4029	35.5899	0.0006[c]
	X_1X_3	15.9996	1	15.9996	20.0482	0.0029[b]
	X_2X_3	4.3887	1	43.887	0.0000	0.9943[ns]
	X_1^2	118.4723	1	118.4723	148.4502	0.0000[c]
	X_2^2	228.9681	1	228.9681	286.9056	0.0000[c]
	X_3^2	3.1221	1	3.1221	3.9121	0.0885[ns]
	Lack of fit	3.0996	2	1.5498	3.1161	0.1322[ns]
	Cor total	849.2212	16			
**Drug Loading Capacity**	Model	589.3865	9	65.4874	11523.0040	0.0000[c]
	X_1	235.8509	1	235.8509	41499.7525	0.0000[c]
	X_2	169.3391	1	169.3391	29796.4992	0.0000[c]
	X_3	0.2132	1	0.2132	37.5176	0.0004[c]
	X_1X_2	30.1607	1	30.1607	5306.9984	0.0006[c]
	X_1X_3	0.7565	1	0.7565	133.1051	0.0000[c]
	X_2X_3	1.0212	1	1.0212	179.6830	0.0000[c]
	X_1^2	0.2299	1	0.2299	40.4498	0.0004[c]
	X_2^2	12.5579	1	12.5579	2209.6569	0.0000[c]
	X_3^2	0.0039	1	0.0039	0.6874	0.4344[ns]
	Lack of fit	0.0023	2	0.0011	0.1533	0.8617[ns]
	Cor total	589.4263	16			

X_1: Finasteride concentration, X_2: Total lipid, X_3: Cholesterol proportion in total lipid, SS: Sum of square; Df: degree of freedom; MS: Mean square. [a] = $p < 0.05$, [b] = $p < 0.01$, [c] = $p < 0.001$, and [ns] = non-significant ($p > 0.05$).
